# Loss-of-function phenotype of a PKCθ^T219A^ knockin mouse strain

**DOI:** 10.1186/s12964-019-0466-8

**Published:** 2019-11-06

**Authors:** Nikolaus Thuille, Kerstin Siegmund, Victoria Klepsch, Jacqueline Schörgenhuber, Sarah Danklmaier, Michael Leitges, Gottfried Baier

**Affiliations:** 1 Department for Pharmacology and Genetics, Medical University Innsbruck, Innsbruck, Austria; 2 Biotechnology Centre of Oslo, Oslo, Norway

**Keywords:** T cell activation, Protein kinase C θ (PKC θ), Thr-219 autophosphorylation site, Interleukin 2 (IL-2) production, NF-κB, NFAT

## Abstract

**Background:**

Protein kinase C θ has been established as an important signaling intermediate in T-effector-cell activation and survival pathways by controlling activity of the key transcription factors NF-κB and NFAT. Previous studies identified an activation-induced auto-phosphorylation site at Thr-219, located between the tandem C1 domains of the regulatory fragment in PKCθ, as a structural requirement for its correct membrane translocation and the subsequent transactivation of downstream signals leading to IL-2 production in a human T cell line.

**Methods:**

The present work aimed to define the role of this phosphorylation switch on PKCθ in a physiological context through a homozygous T219A knockin mouse strain. T cell activation was analyzed by H3-thymidine uptake (proliferative response), qRT-PCR and luminex measurements (cytokine production). NFAT and NF-κB transactivation responses were estimated by Gel mobility shift and Alpha Screen assays. Frequencies of T cell subsets were analyzed by flow cytometry.

**Results:**

Despite a normal T cell development, in vitro activated effector T cells clearly revealed a requirement of Thr-219 phosphorylation site on PKCθ for a transactivation of NF-κB and NFAT transcription factors and, subsequently, robust IL-2 and IFN-γ expression.

**Conclusion:**

This phenotype is reminiscent of the *PKCθ* knockout T cells, physiologically validating that this (p) Thr-219 auto-phosphorylation site indeed critically regulates PKCθ function in primary mouse T cells.

## Background

The protein kinase C (PKC) family consists of 9 members (= isotypes). A few of them are expressed predominantly or at least at particularly high levels in T cells where they have been mapped at the heart of signaling networks that govern proliferation, differentiation and cell survival. PKC isotypes are activated by antigen receptors, costimulatory receptors such as CD28, cytokines and integrins, and their function is regulated by activation of upstream kinases and/or by subcellular localization, which depends on kinase:lipid and kinase:protein interactions, enabling them finally to phosphorylate specific protein substrates [1, 2]. Several members of the PKC family of serine/threonine kinases are crucial in T cell-signaling pathways. Particularly, the classical PKC isotypes, PKCα and PKCβ, and the novel PKC isotypes, PKCθ and η, appear critical for T cell function and play a decisive role in the nature of effector responses [3, 4].

The activity of PKCθ depends on binding to diacylglycerol (DAG) and phosphatidylserine (PS) and is regulated by posttranslational modifications, mainly by auto- and trans-phosphorylation steps on three conserved phosphorylatable serine/threonine residues located at the carboxyl-terminal catalytic domain: Thr-538 (activation loop), Ser-676 (turn motif) and Ser-695 (hydrophobic region) [5]. PKCθ has been shown to translocate to the cell-cell contact site, the so-called immunological synapse (IS), after interaction of a T cell with an antigen-presenting cell (APC) [2]. Both the PI3-K/Vav and ZAP-70/SLP-76 pathways have been implicated in the regulation of PKCθ membrane translocation [6, 7], and the lipid-raft-resident fraction of PKCθ was transiently tyrosine-phosphorylated by Lck on Tyr-90 near the C2-like domain of PKCθ [8]. GLK (germinal center kinase (GCK)-like kinase), a member of the MAP 4 K family, was shown to directly phosphorylate and activate PKCθ at Thr-538 during TCR signaling, as an essential prerequisite for full NF-κB activation [9]. Another elegant study defined the hinge region of PKCθ as a critical structural requirement for localization to the IS via its physical CD28 interaction [10].

Auto-phosphorylation on Thr-219 has been defined by our group as an event essential for correct membrane translocation as well as for a functional transactivation of NF-κB and NFAT pathways and subsequent IL-2 transcription [11]. Previous results were based on overexpression studies in the Jurkat leukemic cell line; here we proposed to test the relevance of this newly defined PKCθ auto-phosphorylation site in a more physiological system. For this purpose, we generated a homozygous T219A knockin mouse, carrying a neutral exchange allele of PKCθ that replaced threonine 219 with an alanine residue, which gave us the possibility to study the biological relevance of Thr-219 auto-phosphorylation site under endogenous conditions in primary mouse T cells.

## Material and methods

### Mice

*PKCθ*^T219A^ mice were generated by Dr. Michael Leitges of The Biotechnology Centre of Oslo, Norway. Briefly, by using recombineering technology, an 11 kb genomic DNA fragment of the PKCθ locus flanked by two homology regions (H1 and H2) was subcloned. Subsequently, an internal fragment containing exon 7 was subcloned on which codon 219 was mutated from ACC to GCC causing an AS exchange from T to A. The modified fragment then got back-recombineered into the targeting vector backbone and finally used for electroporation into ES cells. Subsequently, these mice were breed on a ß-actin promoter-driven Cre transgene background, resulting in a complete NEO cassette deletion.

*PKCθ*^T219A^ mice were born following the expected Mendelian frequency with no differences in growth, weight, viability and fertility. All experiments shown used mice that were backcrossed to C57BL/6 and wild-type littermates as control mice.

All littermates were routinely genotyped by PCR using the primers theta-5′ (GCCTGAACAAGCAGGGTTACCAGTG) and theta-3′ (gacaccacaccctgtttgtttcttcc) to detect the mutant allele (650 bp product) and wild-type allele (539 bp product).

All animals were kept under specific pathogen-free (SPF) conditions. All animal experiments were performed in accordance with the Austrian Animal research act (BGBI. Nr.501/1989 i.d.g.F. and BMWF-66.011/0061- II/3b/2013) and were approved by the Bundesministerium für Wissenschaft und Forschung (bm:wf).

### Analysis of proliferative response and IL-2 cytokine production

CD4^+^ T cells and CD8^+^ T cells were negatively sorted from the spleens and lymph nodes with the MACS CD4+ T Cell Isolation (130–090-860) and MACS CD8+ T Cell Isolation (130–104-075) Kits (Miltenyi Biotec, Bergisch Gladbach, Germany).

For in vitro proliferation, 5 × 10^5^ isolated CD4^+^ and/or CD8^+^ T cells in 200 μl proliferation medium (RPMI supplemented with 10% FCS, 2 mM L-glutamine and 50 units/ml penicillin/streptomycin) were added in duplicate to 96-well plates precoated with anti-CD3 antibody (clone 2C11, 5 μg/ml) and soluble anti-CD28 (clone 37.51, 1 μg/ml; BD Pharmingen) was added. For TCR-independent T cell stimulation, 10 ng/ml phorbol 12,13-dibutyrate (PDBu) and 125 ng/ml of the calcium ionophore ionomycin were added to the media. Cells were harvested on filters after a 48-h stimulation period, pulsed with H3-thymidine (1 mCi/well) in the final 16 h and the incorporation of H3-thymidine was measured with a Matrix 96 direct β counter system.

IL-2 and IFN-γ production in mouse T cells after antibody stimulation was determined by BioPlex technology (BioRad Laboratories) from the supernatant.

### In vitro cell polarization

Naïve CD4^+^ T cells were sorted from the spleens and lymph nodes with the MACS CD4^+^CD62L^+^ T Cell Isolation (30–093-227) Kit (Miltenyi Biotec, Bergisch Gladbach, Germany). Cells were cultured under neutral (TH0) conditions in supplemented IMDM medium in the presence of activating antibodies (5 μg/ml plate-coated anti-CD3 and 1 μg/ml soluble anti-CD28) and iTreg polarizing cytokines: TGF-β [5 ng/ml], human IL-2 [20 ng/ml], αIL-4 [2 μg/ml], αIFN-γ [2 μg/ml] and αIL-12 [2 μg/ml].

Recombinant proteins (recombinant human IL-2 and TGF-β) and blocking antibodies (anti-mouse IL-4, anti-mouse IFN-γ, anti-mouse IL-12) for in vitro cell differentiation were purchased from eBioscience (San Diego, California,USA).

### Western blot analysis

Cells were lysed in ice-cold lysis buffer [5 mM Na3VO4, 5 mM NaP2P, 5 mM NaF, 5 mM EDTA, 150 mM NaCl, 50 mM Tris (pH 7.3), 2% NP-40, 50 μg/ml aprotinin and leupeptin] and centrifuged at 15,000 x g for 15 min at 4 °C. Protein lysates were subjected to immunoblotting using antibodies against actin, DNA polymerase, NFATc1 (all from Santa Cruz Biotechnology), LCK, PKCθ (both from BD Transduction Laboratories), (p) ERK1/2 and ERK (both from Cell signaling). The polyclonal affinity purified (p) Thr-219 PKCθ antibody is from David Biotech.

### Gel mobility shift assays

Nuclear extracts were harvested from 1 × 10^7^ cells according to standard protocols. Briefly, activated CD4^+^ T cells were harvested and washed in PBS and resuspended in 10 mM HEPES (pH 7.9) 10 mM KCl, 0.1 mM EDTA, 0.1 mM EGTA, 1 mM DTT and protease inhibitors. Cells were incubated on ice for 15 min. NP-40 was added to a final concentration of 0.6%, cells were vortexed vigorously, and the mixture was centrifuged for 5 min. The nuclear pellets were washed twice and resuspended in 20 mM HEPES (pH 7.9), 0.4 M NaCl, 1 mM EDTA, 1 mM EGTA, and 1 mM DTT and protease inhibitors, and the tube was rocked for 30 min at 4 °C. After centrifugation for 10 min, the supernatant was collected. Extracted proteins (2 mg) were incubated in binding buffer with [32P]-labeled, double-stranded oligonucleotide probes (AP-1: 5′-CGC TTG ATG ACT CAG CCG GAA-3′; NFAT: 5′-GCC CAA AGA GGA AAA TTT GTT TCA TAC AG-3′) (Nushift; Active Motif). In each reaction, 3 × 10^5^ c.p.m. of labeled probe was used, and the band shifts were resolved on 5% polyacrylamide gels. NFATc1 (Thermo Scientific) and cFos (BD Pharmingen) antibodies were added for super shift reaction. All experiments were performed at least three times with similar outcomes.

### NF-κB -alpha screen assay

Nuclear extracts were prepared as described above and stored at − 70 °C until use.

The assay started with a one-hour incubation step of transcription factor-specific p50 antibody (Santa Cruz X, end concentration 20 μg/ml) and protein A-coated acceptor beads (Perkin Elmer, working concentration 50 μg/ml) in Eppendorf tubes on ice. A following washing step of the acceptor beads in PBS removed excess unbound antibodies. In the meantime, frozen samples were thawed and 1–2.5 μg of protein was incubated with 0.5 ng double stranded biotinylated oligonucleotide probes (NF-κB: 5′-CTG GGG ACT TTC CGC T-3′) in binding buffer (containing 10 mM Tris pH 7.5, 50 mM NaCl, 1 mM DTT, 1 mM EDTA, 5% Glycerol, 0.1% BSA, 1 μg poly dI-dC) on ice in Eppendorf tubes for 30 min to enable formation of transcription factor-DNA complexes (24 μl total volume). Then this protein extract probe mix was transferred to a 384-well microtiter plate, and 3 μl of acceptor beads were added. The plate was covered and incubated on 4 °C in the dark for 30 min. In the meantime, streptavidin-coated donor beads (Perkin Elmer) were prepared (working concentration 50 μg/ml) and finally 3 μl were added to each well. After a final incubation period of 1 hour at room temperature in the dark, the plate was read with a PHERAstar FS multiplate reader [BMG Labtech]. The final concentration of both beads was 20 μg/ml in a total 30 μl reaction volume.

### Flow Cytometry

Single cell suspensions from the spleen, lymph node and thymus were prepared and stained after a washing step for surface marker expression with the following fluorochrome conjugated antibodies: anti-CD3-PECy7, anti-CD4-FITC, anti-CD8-APC and anti-B220-PE, (all from Biolegend). For the staining of activation markers, cells were pre-activated for 24 h with stimulating antibodies (aCD3 and aCD28) and then stained with the following antibodies: anti-CD25-APC, anti-CD44-PECy7 and anti-CD69-PE (all from Biolegend). For analyses of thymocytes the following antibodies were used: anti-CD24-FITC, anti-CD5-PerCP Cy5.5 and TCRβ-Pe Cy7 (all from Biolegend).

For the staining of intracellular FoxP3, the cells were fixed and subsequently permeabilized to the staining of surface antigens. The FoxP3 FITC staining buffer set (eBioscience) was used for the detection of Foxp3. Data were acquired on a FACSCalibur (CellQuest, BD Biosciences) and analyzed with FlowLogic software (eBioscience).

### RNA extraction, cDNA synthesis and real-time quantitative RT-PCR

Total RNA was isolated using the RNeasy Mini Kit (Qiagen) and reverse transcription was performed with the Omniscript Kit (Qiagen) and oligo-dT primers (Promega) according to the manufacturers’ protocols. Gene expression was analyzed by quantitative real-time PCR using TaqMan technology on a 7500/7500 FAST Fast Real-Time PCR instrument (Applied Biosystems). The following reagents were used: 5x QPCR Mix (Rox) from Bio&SELL, TaqMan Gene Expression Assays mouse PKCθ (Mm01340226_m1) and mouse GAPDH endogenous control (4351309) (both Applied Biosystems). All amplifications were conducted in duplicates. GAPDH was used for normalization.

### In vitro suppression assay

CD25^+^CD4^+^ and CD25^−^CD4^+^ T cells were isolated from erythrocyte-depleted cell suspensions of spleens and lymph nodes using the CD4^+^ T cell isolation kit II followed by CD25-PE and anti-PE MicroBeads (all Miltenyi Biotec) according to the manufacturer’s instructions. Sorted CD25^−^CD4^+^ T cells were labeled with 2.5 μM CFSE (Molecular Probes) for 4 min at 37 °C; labeling was stopped by the addition of FCS. T cell-depleted splenocytes (using CD4 and CD8a MicroBeads; Miltenyi Biotec) treated for 45 min with 50 μg/ml mitomycin C (AppliChem) were used, after extensive washing, as antigen-presenting cells. To induce proliferation, 0.5 μg/ml of anti-CD3 (clone 2C-11; BioLegend) was added. 1 × 10^5^ CFSE-labeled CD25^−^CD4^+^ responder T cells were cultured with 1 × 10^5^ APCs in 96-well U-bottom tissue culture plates (Falcon). CD25^+^CD4^+^ T cells were added at the ratios 1 + 1, 1 + 4 and 1 + 9. On day 3 of co-culture, proliferation (based on CFSE dilution) was analyzed by flow cytometry; 7-AAD was added to exclude dead cells from the analysis.

### Ca^2+^ mobilization assay

Isolated primary CD3^+^ T cells (Pan T cell isolation Kit II, Miltenyi Biotec) were incubated for 15 min with 5 μg/ml biotinylated anti-CD3 in PBS at 4 °C. Then the cells were washed and seeded in poly-l-Lysine (Sigma)–coated black-framed clear-bottom 96-well plates (PerkinElmer) at a density of 5 × 10^5^ cells/well in a total volume of 50 μL/well culture medium (RPMI medium with 10% FCS, 2 mM L-glutamine and 50 units/ml penicillin/streptomycin). Ca^2+^ mobilization assays were conducted by using the Fluo-4 Direct Calcium Assay Kit (Invitrogen Life Technologies), according to the manufacturer’s protocol. Briefly, 50 μL of 2× Fluo-4 Direct Calcium Reagent loading solution supplemented with 5 mmol/L probenecid was added to each well and incubated for 1 h at 37 °C.

Assay plates were placed into the PHERAstar FS plate reader (BMG Labtech, Ortenberg, Germany) and changes in intracellular calcium levels were measured in response to TCR activation. The basal fluorescence signal was recorded for 20 s, followed by an addition of 25 μL of Streptavidin dissolved in Fluo-4 Direct Calcium Assay Buffer by means of direct injection and 180 s of continuous recording.

### Statistical analysis

The number of experiments performed are listed in each figure legend. The data were analyzed for statistical significance by one sample unpaired t-test. These statistical analyses were performed with GraphPad Prism software (GraphPad Software Inc.). A *p* value < 0.05 was considered statistically significant. Symbols used in the figures are: * *p* ≤ 0.05, ** *p* ≤ 0.01 and *** *p* ≤ 0.001.

## Results

### T219A mutation alters neither PKCθ protein expression nor mRNA stability and has no effect on T cell development

**Fig. 1:**
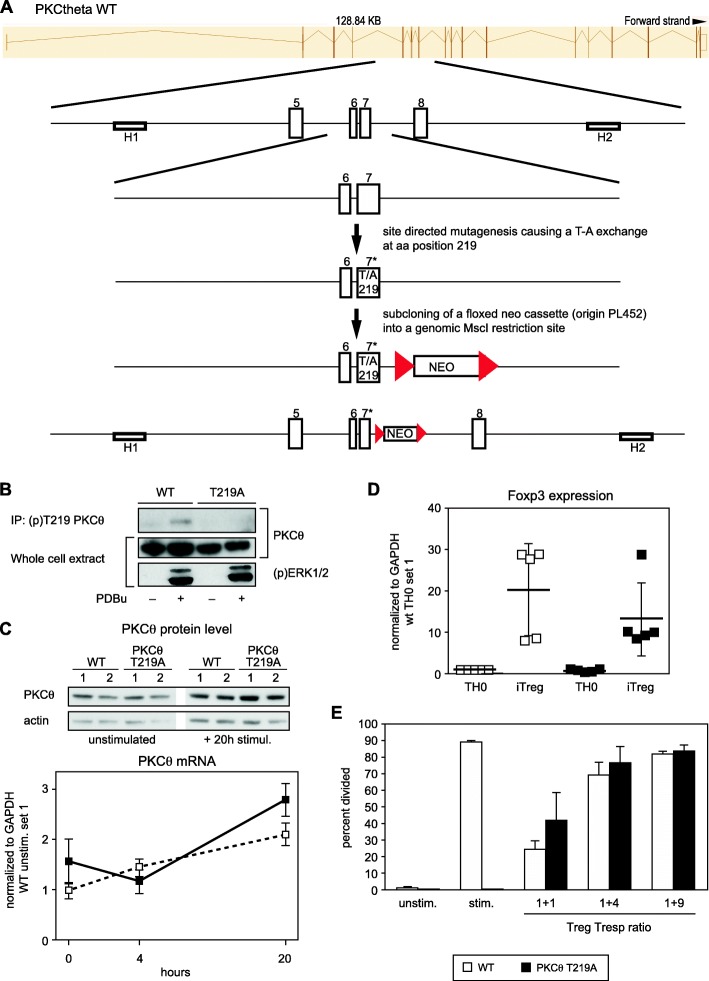
T219A mutation does not alter PKCθ mRNA expression and protein stability. **a** Scheme depicting generation of mutated phosphosite (p) Thr-219. **b** The T219A mutation was biochemically confirmed by immunoblot with lysates of unstimulated and phorbol ester (PDBu) stimulated wild-type and T219A CD3^+^ T cells using our specific (p) Thr-219 PKCθ antibody [David Biotech] for immunoprecipitation and subsequent immunoblot with panPKCθ. Phospho-Erk1/2 staining in the whole cell extract was used to control successful stimulation. **c** The T219A mutation did not alter PKCθ mRNA expression and/or protein stability as verified by RT-PCR and immunoblot (showing the whole cell lysates from two independent experiments, referred as 1 and 2) of unstimulated and CD3/CD28 activated CD3^+^ T cells. RT-PCR data summarizing the results of 3 independent experiments ± SEM are shown. **d** Differentiation of naïve CD4^+^ cells into the iTreg subset was not affected in the knockin mice. Naïve CD4^+^ T cells isolated from wild-type and *PKCθ*^T219A^ mice were differentiated in vitro under neutral conditions (“TH0”: CD3/CD28 only) and iTreg-inducing conditions (IL-2/TGF-β with blocking antibodies against IL-4, IL-12 and IFN-γ) and analyzed for Foxp3 expression by qRT-PCR on day 3 of culture. The house keeping gene gapdh was used for normalization. Data are shown as means ± SEM (*n* = 5). **e** The suppressive capacity of wild-type and T219A CD4^+^CD25^+^ nTreg cells was analyzed in co-cultures with CFSE-labeled CD25^−^CD4^+^ T cells (Tresp) stimulated with APCs and anti-CD3 antibodies. Bar graphs summarizing results of 3 independent experiments are shown. Data are shown as means ± SEM (*n* = 3)

The homozygous T219A knockin strain of mice carrying a specific PKCθ^T219A^ mutant allele (the knockin strategy is depicted in Fig. 1a) were viable, fertile and breed at normal Mendelian ratios. The T219A mutation was confirmed by PCR and immunoblotting of whole cell lysates of unstimulated and stimulated wild-type and T219A CD3^+^ T cells using a specific (p) Thr-219 PKCθ antibody (Fig. 1b). The T219A mutation did not alter PKCθ mRNA expression and/or protein stability as verified by RT-PCR and immunoblot of unstimulated and CD3/CD28 activated CD3^+^ T cells (Fig. 1c).

**Fig. 2:**
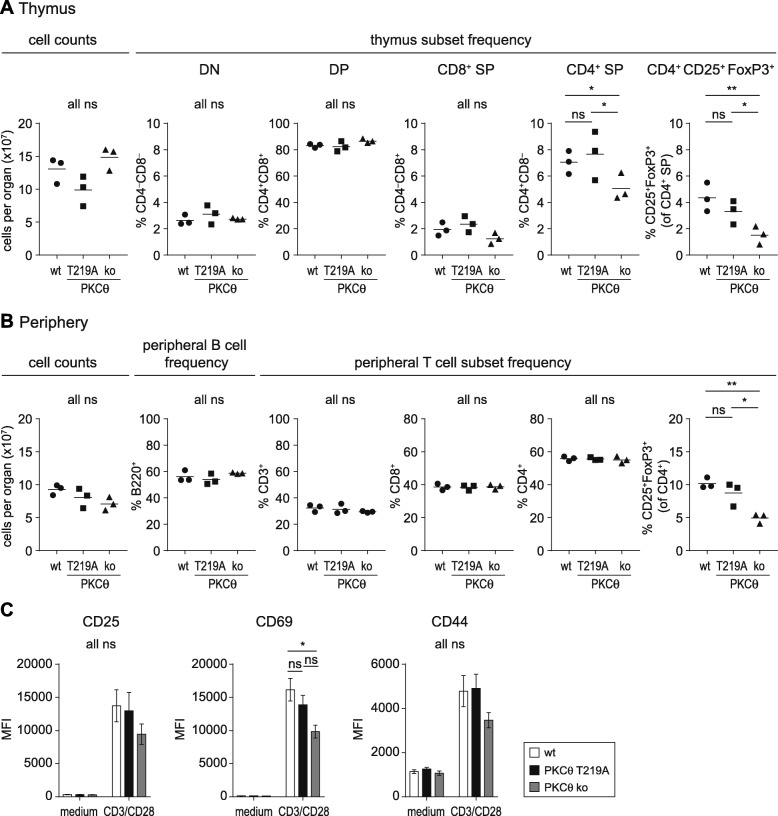
**a** and **b** a detailed flow cytometric analysis of cellularity of thymus and spleen from wild-type, knockin and knockout mice revealed a normal tissue distribution of T and B cells in the T219A knockin mice, demonstrating intact thymocyte development and tissue distribution in the periphery. Total thymocyte and spleen counts were determined ex vivo using a Luna cell counter. Graphs summarizing three experiments are shown (the gating strategy and representative FACS dot blots are shown in Additional file 1: Figure S1). Data are shown as percent positive cells of total lymphocytes. **c**, the surface expression of CD25, CD44, and CD69 on overnight CD3/CD28 activated CD4^+^ T cell populations did not reveal any gross differences between the knockin mice and the wild-type controls. Data are shown as mean fluorescence intensities ± SEM (*n* = 3). Statistical analyses were performed using students t-test

Previous research with PKCθ knockout mice defined a reduced T cell population in the thymus indicating an involvement of PKCθ in the positive selection process during thymocyte development [12, 13]. Flow cytometric analysis of thymocyte populations in wild-type control and *PKCθ*^T219A^ knockin mice revealed no differences in the distribution of CD3, CD4/CD8 double-positive and CD4, CD8 single-positive cells, whereas PKCθ knockout mice showed reduced frequencies of CD4 and CD8 single positive thymocytes (Fig. 2a & Additional file 1: Figure S1), which is in line with previous studies. Furthermore, positive selection and thymocyte maturation, analyzed by CD5/TCRβ and CD24/TCRβ staining, respectively, was comparable between knockin and wild-type control mice (Additional file 2: Figure S2A&B). In addition, the activation dependent upregulation of the positive selection marker CD69 upon overnight stimulation of thymocytes with anti-CD3 was not affected in the knockin setting (Additional file 2: Figure S2C), excluding a possible impact of the (p) Thr-219 site mutation on T cell development. Furthermore, T219A knockin mice showed normal frequencies of T and B cells in secondary lymphoid organs. (Fig. 2b). Examination of the stimulation-dependent upregulation of CD25, CD69 and CD44 surface markers on CD4^+^ (Fig. 2c) subsets revealed no gross differences in the mean fluorescence intensity between in *PKCθ*^T219A^ knockin mice and wild-type controls.

### T219A knockin mice have fully functional CD25^+^Foxp3^+^CD4^+^Treg cells

The activation of conventional T cells upon T cell receptor stimulation critically depends on PKCθ [14, 15]; however, its role in regulatory T (Treg) cell function remains controversial, as some research postulated a negative feedback role of PKCθ for suppressive functions of Tregs [16], whereas other studies provided evidence in support of the dispensability of PKCθ for Treg-mediated suppression [17, 18]. We addressed the role of Thr-219 phosphorylation site on PKCθ in CD25^+^CD4^+^ Treg cell development both in vivo by comparing nTreg frequencies in *PKCθ*^T219A^ and wild-type mice and in vitro by analyzing the FoxP3^+^ expression profile under iTreg polarizing conditions. Flow cytometric analyses revealed no gross difference of Foxp3^+^CD25^+^ CD4^+^ T cells in the thymus and secondary lymphoid organs of *PKCθ*^T219A^ knockin mice (Fig. 2a and b), whereas *PKCθ* knockout mice showed the already published strong reduction in Foxp^3+^CD25^+^CD4^+^ regulatory T cells both in thymus and periphery [17, 18]. The iTreg differentiation assay revealed no differences in the Foxp3 expression profile between polarized CD4^+^ T cells from both of the genotypes, indicating that Thr-219 phosphorylation site on PKCθ is dispensable for iTreg differentiation (Fig. 1d). CD25^+^CD4^+^ nTreg cells isolated from *PKCθ*^T219A^ knockin mice showed comparable suppressive capacities in the in vitro suppression assay: CD25^+^CD4^+^ T cells isolated from T219A mice suppressed the proliferation of activated wild-type CD4^+^ responder T cells to the same degree as CD25^+^CD4^+^ T cells from wild-type mice (Fig. 1e). This is in line with a previous study performed with the *PKCθ* knockout mice [18].

### CD4^+^ and CD8^+^ T cell subsets show an impaired transactivation of the IL-2 effector cytokine

**Fig. 3:**
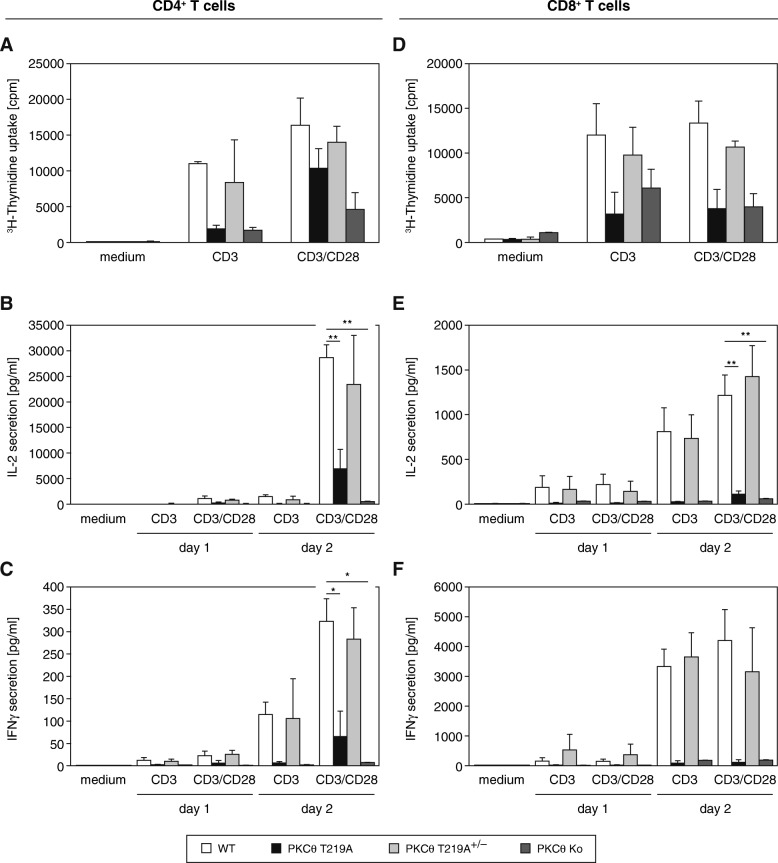
TCR-dependent activation signals lead to a strong defect in IL-2 production both in the peripheral CD4^+^ and CD8^+^ T cell subsets. **a** and **d**, proliferative responses of peripheral MACS-sorted CD4^+^ and CD8^+^ T cells after TCR stimulation revealed a partial defect in the knockin animals similar to responses in *PKCθ*-deficient mice. T cells isolated from heterozygous animals show normal proliferation comparable to the wild-type controls. **b** and **e**, *PKCθ*^T219A^ CD4^+^ and CD8+ T cells show a robust and highly reproducible defect in IL-2 secretion response upon stimulation with CD3/CD28 antibodies, which is reminiscent of the *PKCθ* knockout T cells. C and F IFN-γ levels were reduced both in knockin and knockout T cells whereas the heterozygous genotype showed a mostly unaffected IFN-γ secretion, as revealed by Bioplex measurements. Shown are the mean values of at least three independent experiments ± SEM (**a-f**). Unpaired Students t-test was used for statistics

In contrast to the normal T cell development observed, TCR-induced proliferative responses were partially reduced when T cells express the T219A mutant PKCθ version instead of wild-type PKCθ. Thus, PKCθ^T219A^ T cells show a phenotype similar to the conventional PKCθ-knockout mouse strain. Of note, heterozygous *PKCθ*^T219A^ mutant T cells did not show any effect when compared to wild-type controls (Fig. 3a and d). Notably, both CD4^+^ and CD8^+^ T cell subsets of the T219A knockin mouse line showed a robust and highly reproducible defect in IL-2 secretion responses upon stimulation with CD3/CD28, indicating an important biological role of (p) Thr-219 for PKCθ-dependent IL-2 transactivation processes (Fig. 3b and e). This finding is in line with our previous PKCθ^T219A^ overexpression data defined in the Jurkat cell line [11]. Of note, also activation-induced IFN-γ secretion levels were reduced in T cells lacking PKCθ or expressing the T219A mutated version of *PKCθ* and this defect was similar between both PKCθ-mutant genotypes Fig. 3c and f).

**Fig. 4:**
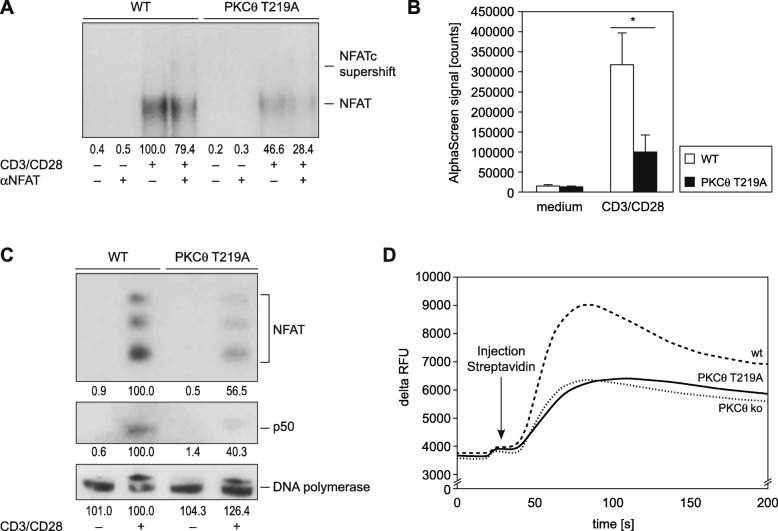
Mutation of (p)T219 on PKCθ leads to NFAT and NF-κB transactivation defects in activated T cells. **a** and **b**, the nuclear extracts of resting and stimulated (overnight) wild-type and *PKCθ*^T219A^ CD4+ T cells were probed for DNA binding to radio-labeled (**a**) or biotinylated (**b**) probes containing NFAT (**a**) and NF-κB (**b**) binding site sequences, as indicated. One representative EMSA experiment of three is shown. The alpha screen measurement shows the summary of four independent NF-κB DNA binding experiments. Data are shown as means ± SEM (*n* = 4). Unpaired Students t-test was used for statistics. **c**, Immunoblots revealed an impaired nuclear import of NFAT and NF-κB transcription factors in activated T219A CD4^+^ T cells. Nuclear extracts of resting and stimulated (overnight) wild-type and T219A CD4^+^ were probed with antibodies against NFAT and the NF-κB subunit p50. DNA polymerase served as the loading control. One representative experiment of three is shown. The Gel shift result (EMSA) and nuclear NFAT and p50 protein levels (immunoblot) were quantified by densitometric analysis. Numbers beneath bands indicate changes compared to stimulated wild-type controls that has been set as 100. **d**, Ca^2+^ mobilization assay revealed an impaired intracellular Ca^2+^ influx upon CD3 crosslinking in mature CD3^+^ from PKCθ^T219A^ knockin and PKCθ knockout mice. One representative experiment of three is shown

In line with the impaired activation-induced cytokine secretion, analysis of the pathways leading to IL-2 transcription revealed reduced binding of NFAT (Fig. 4a) and NF-κB (Fig. 4b) transcription factors to IL-2 promoter-derived DNA enhancer motifs in CD4^+^ T cells upon CD3/CD28 stimulation. Immunoblot analysis of nuclear extracts demonstrated that the weaker DNA binding of NF-κB and NFAT transcription factors is the consequence of reduced nuclear entry of the NF-κB subunit p50 and NFAT upon stimulation (Fig. 4c). It has previously been described that PKCθ is required for intracellular Ca^2+^ mobilization and subsequently downstream calcineurin and NFAT transactivation [15]. Given the strong reduction of TCR-induced NFAT nuclear entry in *PKCθ*^T219A^ derived T lymphocytes we analyzed how the *PKCθ*^T219A^ mutant is also able to regulate intracellular Ca^2+^ capacities. The TCR activation of Fluo-4-loaded mature CD3^+^ purified from spleen and lymph nodes of PKCθ ^T219A^ knockin mice led to a reduced cytosolic Ca^2+^ increase when compared to wild-type control T lymphocytes (Fig. 4d). This defect resembles the *PKCθ* knockout phenotyp and implicates a function of Thr-219 site in Ca^2+^ mobilization. The strong defect in the IL-2 transactivation pathway, namely NF-κB and NFAT nuclear entry, is reminiscent of the *PKCθ* knockout phenotype [15], indicating that the Thr-219 phosphorylation site plays a major role in these critical T cell activation processes.

## Discussion

The central role of PKCθ in T cell activation and survival processes is well established by findings in PKCθ loss of function mouse strains, revealing that mature *PKCθ*-deficient peripheral T cells display impaired IL-2 cytokine production in response to TCR/CD28 co-stimulation, mainly by affecting AP-1, NF-κB and Ca2^+^/NFAT signaling pathways [14, 15, 19, 20]. The signals triggered by the T cell receptor and CD28 costimulatory molecules induce important auto- and trans-phosphorylation events in conserved serine/threonine residues [Thr-538, Ser-676, Ser-695] [9, 21, 22] or tyrosine residue [Tyr-90) [8, 23] in the catalytic domain of PKCθ which are essential pre-requisites for kinase activation of PKCθ. In addition, a structural requirement of the Pro-rich motif in the V3 domain of PKCθ has been shown to be essential for a proper recruitment in the central supramolecular activation cluster of the IS and PKCθ-CD28 complex formation [10]. Recently a study addressed the relevance of the N-terminal variable domain V1 (which is encoded by exon 2) for PKCθ function via the use of a mouse line carrying the mutated version of exon 2 (PKCθ-E2mut). PKCθ-E2 mutation led to impaired T cell development in vivo and defective early activation responses of mature T cells, showing a phenotype similar to conventional PKCθ-deficient mice [24].

Phosphorylation on Thr-219 has been defined by our research team to be critical for proper NF-κB and NFAT as well as subsequent IL-2 promoter transactivation in Jurkat cells upon anti-CD3/CD28 co-stimulation [11].

A critical re-evaluation of our previous findings in a physiological setting, employing primary T cells of a homozygous *PKCθ*^T219A^ mutant mouse strain was the starting point of our recent work. Isolated primary T cells of this knockin mice showed normal endogenous PKCθ^T219A^ expression levels comparable to those in wild-type mice, indicating that T219A mutation does not affect PKCθ gene expression and protein stability. The activation-dependent phosphorylation of PKCθ on Thr-219 was confirmed in phorbol ester (and CD3/CD28, data not shown) stimulated murine wild-type T cells (Fig. 1b) via the use of a Thr-219 phosphorylation site-specific antibody; the knockin-derived T cells served as negative control.

Thr-219 is located in the C1 domain of the regulatory fragment in PKCθ, which has been described to contain a binding site for DAG or non-hydrolysable analogues called phorbol esters. Of note, this domain is fully capable of binding DAG in both wild-type and T219A knockin setting, as previously established [11]. Consistently, membrane translocation upon CD3/CD28-stimulation or phorbol ester treatment is not impaired in the mutant PKCθ^T219A^ protein in primary murine CD3^+^ T cells, when tested by biochemical subcellular fractionation assay (unpublished data). However, these data do not directly rule out any disturbed localization of mutant PKCθ^T219A^ protein to specific functional membrane compartments (rafts and/or I-synapse).

Since it has been reported that PKCθ deficiency affects the positive selection process in thymocyte development, leading to a lower thymic frequency of CD4 and CD8 single positive cells [12, 13, 18], we carefully checked if there are any abnormalities within the T cell compartment of *PKCθ*^T219A^ mice: our results clearly show no differences in T cell subset numbers and frequencies in thymus and periphery between wild-type control and knockin mice. Furthermore, the expression of thymic selection and maturation markers CD5, CD69 and CD24 were indistinguishable between wild-type and knockin animals.

In line with previous studies [18, 24] we observed reduced frequencies of Foxp3^+^CD25^+^CD4^+^ natural regulatory T cells in the thymus and also peripheral lymphoid organs of mice lacking PKCθ. In contrast, T219A knockin mice show normal distribution of Treg cells both in thymus and secondary lymphoid organs resembling the wild-type phenotype.

When we analyzed the proliferative and secretory responses of mature T cells, we found a significant activation defect in CD3/CD28-stimulated CD4^+^ and CD8^+^ T cells of the knockin mouse line when compared to wild-type sibling controls. This impairment is secondary to disturbed downstream signaling pathways as the transactivation of NF-κB and NFAT transcription factors was considerably affected by the T219A mutation on PKCθ. These findings are in line with our previous data from Jurkat cell transfection assays and indicate that the *PKCθ*^T219A^ mutant T cells are a phenocopy of the *PKCθ* knockout cells [14, 15].

Interestingly and when directly comparing thymocytes derived from T219A knockin versus knockout strategies, our data reveal a selective phenotype difference in thymocytes (Fig. 2a & Additional file 1: Figure S1 & Additional file 2: Figure S2) but not in peripheral T cells (Figs. 3 and 4), derived from these distinct genetic PKCθ LOF approaches. This intriguing issue needs to be addressed in future studies.

## Conclusion

In summary, the phenotype of mature T cells derived from this PKCθ^T219A^ knockin mouse strain - as a distinct genetic loss-of-function approach - resembles mostly the *PKCθ* knockout immune phenotype. In contrast to PKCθ knockout T cells, and despite bearing a single amino acid substitution, PKCθ^T219A^ is still expressed at physiological protein levels. Thus, it provides an independent confirmation of the critical PKCθ function in early T cell activation. Furthermore, our data show that the Thr-219 phosphorylation site on PKCθ plays a major functional role in T cell activation processes in the effector T cell compartment. As such, a detailed analysis of this (p) T219 site within the PKCθ protein to specifically delineate its detailed mode of action needs to further unravel the complex activation steps of PKCθ in future studies.

## Supplementary information

**Additional file 1: Figure S1**. A, FACS dot plots depicting the gating strategy used for analyzing the thymic subsets shown in Fig. 2a. B, Representative FACS dot blots showing thymocyte subsets of all three genotypes (wild-type, PKCθ^T219A^ knockin and PKCθ knockout mice).

**Additional file 2: Figure S2.** Examination of thymic positive selection via Flow cytometry reveals a normal thymocyte maturation in PKCθ^T219A^ mice. A, FACS analysis of TCRβ/CD24 profile on CD4 and CD8 SP thymocytes showed no defect in thymocyte maturation in the T219A knockin mice. Graphs summarizing three experiments are shown. B, Further examination of the thymic positive selection process via analysis of the CD5 marker was also showing no differences in pre- and post-positive selection populations between knockin and wild-type control mice. The different populations can be distinguished by a specific distribution (rearrangement) of both the TCRβ and CD5 marker: TCRβ^lo^CD5^lo^ (pre-positive selection population), TCRβ^lo^CD5^int^ (cells initiating positive selection), TCRβ^int^CD5^hi^ (cells undergoing positive selection process) and TCRβ^hi^CD5^hi^ (post-positive selection population). Representative FACS dot blots are shown. C, Expression of CD69 on ex vivo stimulated thymocytes (via anti-CD3 cross linking over night) was comparable between the knockin mice and the wild-type controls. Data are shown as mean fluorescence intensities ± SEM (*n* = 3). Statistical analyses were performed using students t-test.

## Data Availability

All data used in this study are available from the corresponding author on reasonable requests.

## Abbreviations

APC: Antigen-presenting cell
DAG: Diacylglycerol
IL-2: Interleukin-2
IS: Immunological synapse
NFAT: Nuclear factor of activation in T cells
NF-κB: Nuclear factor κ B
PDBu: Phorbol 12,13-dibutyrate
PKC: Protein kinase C
PS: phosphatidylserine
TCR: T cell receptor
