# Supplementary material for: Loss-of-function phenotype of a PKCθT219A knockin mouse strain
Source: Cell Commun Signal. 2019 Nov 6;17:141. doi: 10.1186/s12964-019-0466-8 (PMC6836476; doi:10.1186/s12964-019-0466-8)
Supplement: Supplementary file 1 [file 12964_2019_466_MOESM1_ESM.docx]

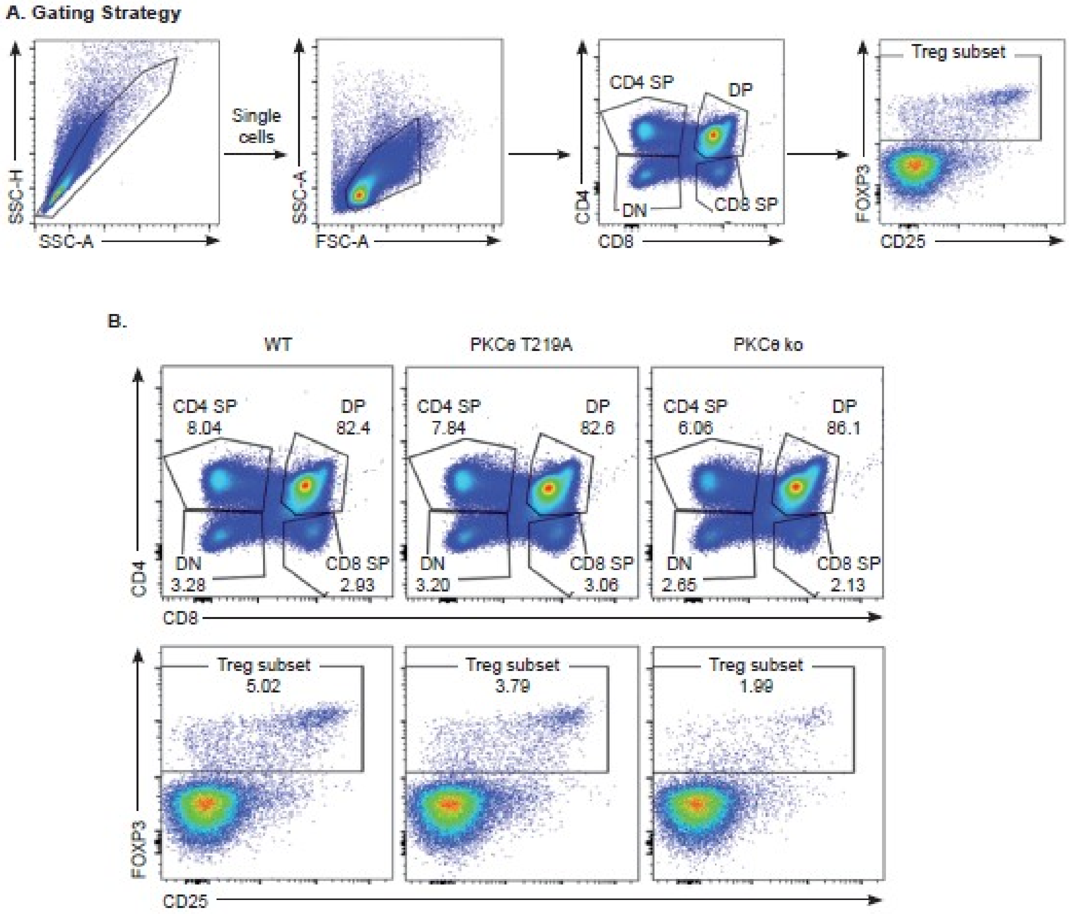
A. Gating Strategy
SSC-H
SSC-A
Single cells
SSC-A
FSC-A
CD4 SP
DP
CD4
DN
CD8 SP
CD8
Treg subset
FOXP3
CD25
B.
WT
PKCθ T219A
PKCθ ko
CD4 SP
8.04
DP
82.4
CD4 SP
7.84
DP
82.6
CD4 SP
6.06
DP
86.1
DN
3.28
CD8 SP
2.93
DN
3.20
CD8 SP
3.06
DN
2.65
CD8 SP
2.13
CD4
CD8
Treg subset
5.02
Treg subset
3.79
Treg subset
1.99
FOXP3
CD25
