# Supplementary material for: Loss-of-function phenotype of a PKCθT219A knockin mouse strain
Source: Cell Commun Signal. 2019 Nov 6;17:141. doi: 10.1186/s12964-019-0466-8 (PMC6836476; doi:10.1186/s12964-019-0466-8)
Supplement: Supplementary file 2 [file 12964_2019_466_MOESM2_ESM.docx]

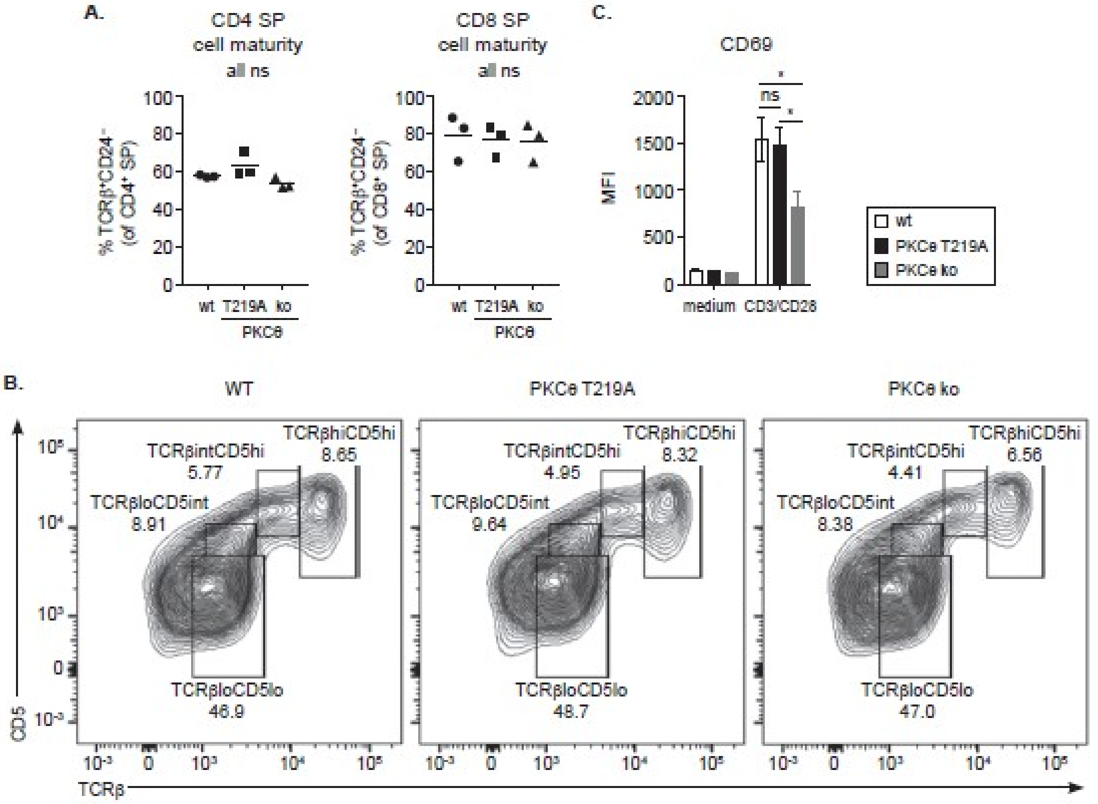
A.
CD4 SP
cell maturity
all ns
% TCRβ+CD24−
(of CD4+ SP)
wt
T219A
ko
PKCθ
CD8 SP
cell maturity
all ns
% TCRβ+CD24−
(of CD8+ SP)
C.
CD69
MFI
medium
CD3/CD28
ns
wt
PKCθ T219A
PKCθ ko
B.
WT
PKCθ T219A
PKCθ ko
TCRβintCD5hi
5.77
TCRβhiCD5hi
8.65
TCRβloCD5int
8.91
TCRβloCD5lo
46.9
TCRβintCD5hi
4.95
TCRβhiCD5hi
8.32
TCRβloCD5int
9.64
TCRβloCD5lo
48.7
TCRβintCD5hi
4.41
TCRβhiCD5hi
6.56
TCRβloCD5int
8.38
TCRβloCD5lo
47.0
CD5
TCRβ
